# Diet and glycaemia: the markers and their meaning. A report of the Unilever Nutrition Workshop

**DOI:** 10.1017/S0007114514003547

**Published:** 2014-12-11

**Authors:** Marjan Alssema, Hanny M. Boers, Antonio Ceriello, Eric S. Kilpatrick, David J. Mela, Marion G. Priebe, Patrick Schrauwen, Bruce H. Wolffenbuttel, Andreas F. H. Pfeiffer

**Affiliations:** 1 Unilever R&D, Olivier van Noortlaan 120, Vlaardingen, The Netherlands; 2 EMGO Institute for Health and Care Research, VU Medical Center, Amsterdam, The Netherlands; 3 Insititut d'Investigacions Biomèdiques August Pi i Sunyer (IDIBAPS), Barcelona, Spain; 4 Department of Clinical Biochemistry, Hull York Medical School, Hull, UK; 5 Center for Medical Biomics, University Medical Center Groningen, Groningen, The Netherlands; 6 Department of Human Biology, NUTRIM School for Nutrition, Toxicology and Metabolism, Maastricht University Medical Center, Maastricht, The Netherlands; 7 Department of Endocrinology, University of Groningen, University Medical Center Groningen, Groningen, The Netherlands; 8 Department of Endocrinology, Diabetes and Nutrition, Charité Universitätsmedizin Berlin, Campus Benjamin Franklin, Berlin, Germany; 9 Department of Clinical Nutrition, German Institute of Human Nutrition, Potsdam, Germany

**Keywords:** Diet, Glycaemia, Glycated Hb, Fructosamine

## Abstract

Consumption of carbohydrate-containing foods leads to transient postprandial rises in blood glucose concentrations that vary between food types. Higher postprandial glycaemic exposures have particularly been implicated in the development of chronic cardiometabolic diseases. Reducing such diet-related exposures may be beneficial not only for diabetic patients but also for the general population. A variety of markers have been used to track different aspects of glycaemic exposures, with most of the relevant knowledge derived from diabetic patients. The assessment of glycaemic exposures among the non-diabetic population may require other, more sensitive markers. The present report summarises key messages of presentations and related discussions from a workshop organised by Unilever intended to consider currently applied markers of glycaemic exposure. The particular focus of the meeting was to identify the potential applicability of glycaemic exposure markers for studying dietary effects in the non-diabetic population. Workshop participants concluded that markers of glycaemic exposures are sparsely used in intervention studies among non-diabetic populations. Continuous glucose monitoring remains the optimal approach to directly assess glycaemic exposure. Markers of glycaemic exposure such as glycated Hb, fructosamine, glycated albumin, 1,5-anhydroglucitol and advanced glycation end products can be preferred dependent on the aspect of interest (period of exposure and glucose variability). For all the markers of glycaemia, the responsiveness to interventions will probably be smaller among the non-diabetic than among the diabetic population. Further validation and acceptance of existing glycaemic exposure markers applied among the non-diabetic population would aid food innovation and better design of dietary interventions targeting glycaemic exposure.

## Introduction (Marjan Alssema)

Consumption of carbohydrate-containing foods leads to varying levels of transient postprandial rises in blood glucose. Higher levels of postprandial glycaemic exposures have been implicated in the development of chronic cardiometabolic diseases, particularly type 2 diabetes mellitus (T2DM) and CVD^(^ ^1^ ^)^. Patients with T2DM are particularly exposed to higher blood glucose levels, due to both fasting glucose and postprandial glucose excursions. Recent guidelines from the International Diabetes Federation advise the active treatment of postprandial glucose with pharmacological therapy as well as dietary intervention in these patients^(^ ^2^ ^)^. Although much less evidence is available for the non-diabetic population, there is broad consensus that reducing the exposure to high postprandial glucose concentrations is beneficial for their health too^(^ ^3^ ^)^.

Glycated Hb (HbA1c) is the most accepted marker to evaluate the longer-term blood glucose control in diabetic patients. However, there are hardly any data on the use of HbA1c or other glycaemic exposure markers in (dietary) intervention trials among non-diabetic individuals, i.e. those with normal or impaired glucose tolerance (IGT) who experience lower and more sustained glycaemic exposure. Diets can have an impact on postprandial glucose concentrations also in the non-diabetic population; however, it is unclear whether current markers are sensitive to (smaller dietary) changes in glycaemic exposure. Such information would be particularly relevant to evaluating the efficacy of dietary and lifestyle approaches on glycaemic exposure in the general population and its role in reducing disease risks.

To address this challenge, Unilever organised a workshop with experts on diet and glycaemia on 16–17 December 2013, in Vlaardingen, The Netherlands. The workshop was attended by scientists from academia and Unilever. The present report summarises the key messages of the presentations held and related discussions among the wider audience. On the first workshop day, we focused on the relevance of postprandial glucose lowering and comprised presentations on the food industry perspective, the physiological consequences of postprandial glucose lowering, and the underlying mechanisms leading to postprandial glycaemic exposure. On the 2nd day, we addressed how glycaemic exposure can be measured. The presentations included the characteristics (glucose variability) and use of different glycaemic exposure markers. Thereafter, the knowns and unknowns of the applicability of glycaemic exposure markers in the non-diabetic population were discussed and summarised. The ultimate objectives of the meeting were to review present knowledge and consensus about the characteristics of currently applied markers of glycaemic exposure and the applicability of such markers among the non-diabetic population.

## The food industry perspective on postprandial glucose lowering (David Mela)

Most large global food manufacturers have stated goals for monitoring and improving the nutritional quality of their products and portfolio. For example, under the Unilever Sustainable Living Plan, Unilever has set out a range of specific nutrition targets for improving the health and well-being of consumers^(^ ^4^ ^)^. Given the increasing burden of T2DM worldwide, there is a growing interest to see where manufactured foods can make a contribution to reducing the prevalence and risks of this disease. Unilever as well as other food manufacturers are therefore looking at how dietary approaches might help in reducing glycaemic exposures, and in particular reduce postprandial glucose (and insulin) responses.

Food industry approaches to the delivery of health benefits differ in important ways from pharmaceutical approaches. Most importantly, diet would always be seen as one contributor to a healthier lifestyle and reduced disease risks, but individual foods will never be claimed to treat, prevent or cure disease. Individual foods can, however, have beneficial physiological effects that may be linked to a potential for improving public health in the general population, and can have a wide reach in terms of numbers of consumers and consumption occasions. While individuals with diabetes and IGT may not be the specific target group for such products, these groups that in particular experience hyperglycaemia have a significant and growing presence within the general consumer population.

The reduction of glycaemia is generally endorsed by experts as a health-relevant target for dietary intervention^(^ ^2^ ^,^ ^3^ ^)^. This view is also reflected in regulatory views on product health claims. The European Food Safety Authority has stated that the reduction of postprandial glycaemia may be a beneficial physiological effect, provided postprandial insulin is not disproportionally increased^(^ ^3^ ^)^. Evidence for the effects of foods on postprandial glucose and insulin concentrations is derived from acute testing, typically involving single exposures and monitoring of measures for 2–3 h postprandially. The European Food Safety Authority furthermore cites the ‘maintenance’ of normal blood glucose concentrations as a beneficial physiological effect. The required substantiation for such an effect is an improvement in HbA1c over a period of 12 weeks, while fasting and post-load glucose as well as fructosamine are regarded as ‘supportive evidence’. The scientific evidence underpinning the suggested health relevance of postprandial glucose lowering is largely derived from studies with gut-active enzyme inhibitors (e.g. acarbose, miglitol)^(^ ^5^ ^,^ ^6^ ^)^, the effects of postprandial glucose-lowering diets^(^ ^7^ ^,^ ^8^ ^)^ and observational data on lower-glycaemic index (GI) or load diets^(^ ^9^ ^,^ ^10^ ^)^.

**Fig. 1:**
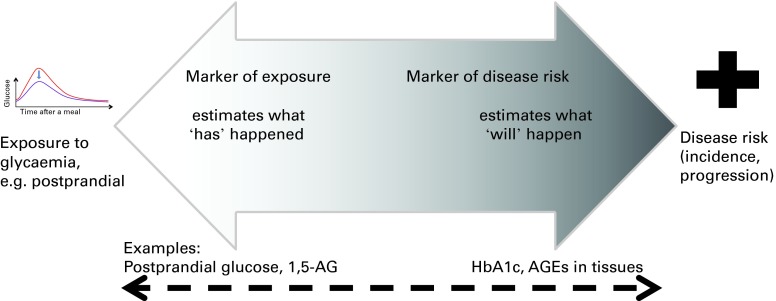
Markers of glycaemic exposure and markers of disease risk are the estimates of different reference points. The same marker can be a reflection of both exposure and disease risk. Postprandial glucose and 1,5-anhydroglucitol (1,5-AG) are examples of the markers of recent glycaemic exposure. Glycated Hb (HbA1c) and advanced glycation end products (AGE) in tissues are the markers of chronic, but not recent, exposure and are also the accepted markers of disease risk. A colour version of this figure can be found online at journals.cambridge.org/bjn

The use of HbA1c as the accepted marker to evaluate blood glucose exposure stems from its use in clinical practice to monitor glycaemic control in diabetic patients. HbA1c is recognised as a marker of future risk of diabetes complications, and many intervention and epidemiological studies have supported its relationship to micro- and macrovascular complications^(^ ^11^ ^)^. Furthermore, since HbA1c is accepted as a diagnostic criterion for diabetes, it is also a direct measure of diabetes risk. However, accepted markers of disease risk (such as HbA1c) may not necessarily be the most appropriate for assessing glycaemic exposure for a given research setting, depending on the objectives, population of interest, timings, costs, etc. (Fig. 1). HbA1c is a marker reflecting long-term glycaemic control (3 months), not shorter-term responses (weeks, days or hours). Unfortunately, at present, there is limited endorsement for any specific markers of (diet-induced) glycaemic exposures between acute (postprandial glucose) and 3 months (HbA1c). In addition, among non-diabetic populations, there is relatively little information on the use of HbA1c and other glycaemic exposure markers. Academic and industrial research and food innovation will benefit from expert-endorsed markers appropriate for the general population, which can be used for evaluating, substantiating and communicating product benefits.

## The role of postprandial glucose and insulin in the pathophysiology and onset of diabetes (Andreas F. H. Pfeiffer)

It is established that increased postprandial glucose precedes the development of type 2 diabetes as identified by the oral glucose tolerance test as a diagnostic criterion of IGT. However, there is no strong evidence for a pathophysiological role of postprandial glucose in the development of T2DM in the absence of insulin resistance or β-cell dysfunction in a healthy organism. High-GI diets increased insulin resistance as measured by homeostasis model assessment values in the DIOGenes study^(^ ^12^ ^)^, but showed variable associations with the risk of diabetes^(^ ^9^ ^)^. In particular, in observational studies where the assessment of GI/glycaemic load is done by a FFQ, and where (residual) confounding of, for example, fibres may play a role, the interpretation of such findings remains challenging. Moreover, the GI was variably associated with intima–media thickness as a predictive marker of atherosclerosis and cardiovascular events in controlled studies^(^ ^13^ ^)^. There is evidence that acarbose reduces the incidence of T2DM in patients with IGT^(^ ^5^ ^)^; however, it is unclear whether this should be seen as prevention, delay or masking of diabetes.

In established T2DM, elevated glucose concentration is clearly related to the development of micro- and macrovascular complications. However, it is unresolved whether the risk of complications is more related to postprandial glucose and the amplitude of glucose excursions or to average glucose as measured by HbA1c. While numerous biomarkers are clearly increased depending on postprandial glucose excursions, their relationship to disease risk is debated. Regarding macrovascular complications, studies supporting both views have been presented, such as the Nateglinide and Valsartan in Impaired Glucose Tolerance Outcomes Research study^(^ ^14^ ^)^ and the Diabetes Intervention Study from Dresden^(^ ^15^ ^)^. A Cochrane systematic review and meta-analysis of α-glucosidase inhibitors in type 2 diabetic patients has shown that α-glucosidase inhibitors have a clear beneficial effect on glycaemic control and post-load insulin levels but not on plasma lipids^(^ ^6^ ^)^. A positive effect of lowering postprandial glucose by acarbose has also been shown on atrial natriuretic peptide, a biomarker indicating beneficial effects on the heart^(^ ^16^ ^)^. As a natriuretic peptide, this is one of the vascular protective agents, and this hormone is known to decrease during an oral glucose tolerance test. Since HbA1c depends on both average fasting and postprandial glucose concentrations, both components are likely to contribute. Postprandial glucose metabolism plays an important role in hepatic fat accumulation^(^ ^17^ ^)^ and inflammatory responses^(^ ^18^ ^,^ ^19^ ^)^.

A group that is likely to profit significantly from good control of postprandial glucose and nutritional interventions are obese people with the metabolic syndrome and large excursions of insulin and postprandial glucose after meals. Among individuals with IGT, nutrition therapy clearly provides powerful tools to prevent progression to diabetes, as has been shown in the Diabetes Prevention Study and Diabetes Prevention Program^(^ ^20^ ^,^ ^21^ ^)^. Moreover, dietary interventions using high total fat based on low-carbohydrate, high-MUFA and PUFA strategies (Mediterranean diet) have been shown to reduce cardiovascular events without weight loss or exercise^(^ ^22^ ^)^. A third approach consists of the use of increased protein contents, which improves postprandial glucose by accelerating and augmenting fast insulin secretion while reducing overall insulin requirements^(^ ^23^ ^)^. This may also be achieved by high-protein snacks preceding meals^(^ ^24^ ^)^. Protein from plant sources appears to differ from animal protein, particularly red or processed meat^(^ ^25^ ^)^. Diets high in animal protein are associated with an increased risk of diabetes, which is not the case for diets high in plant protein^(^ ^25^ ^)^. Since many people avoid plant protein due to associated soluble fibres in legumes and intestinal discomfort, there is much potential for the development of healthy foods based on plant protein. Further components of great interest are dietary fibres and whole grains. Primarily non-soluble cereal fibres have been associated with a decreased risk of diabetes^(^ ^26^ ^)^ and CVD^(^ ^27^ ^)^. Findings from prospective cohort studies showed that whole grain intake is inversely associated with the risk of T2DM, and this association is stronger for bran than for germ^(^ ^26^ ^)^. A hypothesis could be that cereal fibre intake is associated with higher insulin sensitivity, which has been confirmed in the Framingham Offspring Study^(^ ^28^ ^)^ and in a recent human intervention trial^(^ ^29^ ^)^. These foods would be attractive for people aiming at a healthy lifestyle and for those with diabetes or prediabetes.

In conclusion, the evidence for a health benefit of reducing glycaemic exposure is so far not conclusive, in particular for the general ‘healthy’ population. Studies with GI-lowering diets provide some evidence, but the interrelationship with fibres and resistant starch makes them difficult to interpret. Large intervention trials with acarbose in (pre)diabetes do provide the strongest evidence. Studies on the biomarkers of heart, liver function and inflammation have suggested that the metabolic effects of postprandial glucose-lowering interventions may be physiologically broad and require further study.

## Metabolic routes underlying postprandial glycaemia (Hanny M. Boers and Marion G. Priebe)

Postprandial glycaemia is not only determined by glucose influx from food (exogenous glucose), but also by glucose production by the liver (endogenous glucose) and glucose uptake in tissues (disposal). For reliable estimation of the different factors that determine the postprandial glucose response (influx, production and disposal), the dual-label isotope technique can be used by labelling the starch in the food products with ^13^C (influx) and infusing the volunteers with a tracer amount of d-[6,6-^2^H_2_]glucose (disposal)^(^ ^30^ ^–^ ^32^ ^)^.

As shown by isotope labelling studies, starch consumption can have an impact on the glycaemic response by affecting glucose influx^(^ ^33^ ^)^, glucose disposal^(^ ^33^ ^,^ ^34^ ^)^ or endogenous glucose production^(^ ^35^ ^)^, or by a combination of these^(^ ^33^ ^)^. Therefore, the overall blood glucose response is not the same as starch digestibility, the latter varying widely between foods (even within one food group). Starch digestibility can be predicted by an *in vitro* assay^(^ ^36^ ^)^ and is often assumed to be the basis for variation in GI. However, this is inherently wrong, because the GI is determined not only by starch digestibility but also by glucose production and disposal.

Recent studies have shown that glycaemic responses are achieved by fluctuations in more than one factor. Priebe *et al.* ^(^ ^35^ ^)^ showed that a lower glycaemic and insulin response of wheat bread during the first 2 h compared with glucose in water was not due to a lower glucose influx from bread, but to a lower endogenous glucose production in the liver. Eelderink *et al.* ^(^ ^37^ ^)^ showed that glycaemic response was the same for bread and pasta, but influx of glucose was lower for pasta than for bread (30 %) as was glucose disposal^(^ ^33^ ^)^.

In conclusion, the different metabolic routes by which foods can have an impact on the overall blood glucose response must be taken into account for a mechanistic understanding of food impacts on glycaemia.

## Markers of glucose variability and postprandial peaks (Antonio Ceriello)

There are indications that glycaemic variability, beyond HbA1c, has an impact on the development of diabetes complications^(^ ^38^ ^,^ ^39^ ^)^. The overall glycaemic exposure as reflected by HbA1c does not necessarily reflect the glycaemic variability that an individual is exposed to. For example, from continuous glucose monitoring (CGM) studies in type 1 diabetes, it is known that even patients in good glycaemic control can experience wide glucose fluctuations^(^ ^40^ ^)^. Glycaemic variability over the day (in contrast to between days) is importantly determined by meal intake and physical exercise. Oxidative stress is the putative link between glycaemic variability and endothelial dysfunction, ultimately leading to diabetic complications^(^ ^39^ ^)^. Although the precise mechanism is not completely resolved, there are indications from clinical studies that a high degree of glycaemic variability (glucose oscillations), more than chronic hyperglycaemia, leads to oxidative stress and endothelial dysfunction^(^ ^41^ ^,^ ^42^ ^)^. These effects have been shown during spontaneous glucose oscillations in T2DM patients as well as during induced glucose oscillations in both normal glucose-tolerant individuals and diabetic patients^(^ ^41^ ^,^ ^42^ ^)^.

Although a certain degree of variability has also been observed in subjects with normal glucose tolerance, glucose variability increases in individuals with diabetes and in those with impaired blood glucose regulation (impaired fasting glucose and IGT). Therefore, it becomes crucial not merely to identify the boundary beyond which glucose variability takes on a pathological meaning, but, more importantly, to better define the concept of glucose variability. A clear consensus on the gold-standard method to measure glucose variability in clinical practice and research is still lacking, although a number of indicators have been proposed^(^ ^43^ ^)^. Some of the most frequently used methods are reviewed below. Many of the current marketed CGM devices provide more than one of these estimates of glycaemic variability, and normative values have now been described^(^ ^44^ ^)^.

### Postprandial glucose, mean blood glucose and standard deviation

Relatively straightforward measures of glucose variability include the direct measurement of postprandial glycaemia, the mean blood glucose level and its standard deviation. Postprandial hyperglycaemia can be measured simply by monitoring postprandial glycaemia 1–2 h after meals.

Standard deviation, an index of the dispersion of data around mean blood glucose, was initially viewed as the simplest approach for the evaluation of glucose variability, beyond the determination of mean blood glucose. It has very recently been observed that it is only in type 1 diabetes mellitus that there is a linear relationship between HbA1c (an expression of the mean plasma concentration of glucose) and glucose variability, and this is particularly the case in subjects with higher HbA1c levels^(^ ^45^ ^)^. The authors concluded that these findings indicate that reducing glycaemic variability in patients with poor glycaemic control can help improve HbA1c.

### J index and CV

The imperfect relationship between mean blood glucose and standard deviation can be partly resolved using indices that correct the standard deviation for mean blood glucose, such as the CV, i.e. the relationship between standard deviation and the absolute value of the arithmetic mean glycaemia or the *J* index^(^ ^46^ ^)^. One of the limits and therefore one of the criticisms of the use of standard deviation and related indices is that standard deviation considers all the excursions without, however, giving a different weight to major and minor swings.

### Mean amplitude of glucose excursion

The mean amplitude of glucose excursion is the mean of the daily glucose excursions that exceed the standard deviation measured over the 24 h period. It is based on the use of continuous blood glucose monitoring over 24 h or, albeit with certain reserves, on complete seven-point blood glucose profiles to calculate the mean and standard deviation, and considers only major glucose excursions^(^ ^47^ ^)^. The mean amplitude of glucose excursion is probably one of the most applied indices of glucose variability.

### Continuous overlapping net glycaemic action

The recently introduced continuous overlapping net glycaemic action (CONGA) index is an indicator of within-day glucose variability^(^ ^48^ ^)^. After the first number of hours of observation, obtained by means of CGM, it calculates the difference between current observation and observation in the previous hours. CONGA is defined as the standard deviation of the recorded differences. The higher the CONGA value, the greater the glycaemic excursion. The frequently used parameters, CONGA1, CONGA2 and CONGA4, coincide with observations lasting 1, 2 or 4 h, and they are, therefore, expressions of glucose variability within these intervals.

### Low blood glucose index, high blood glucose index, average daily risk range and blood glucose risk index

These parameters were developed by Kovatchev starting from 1998, as a logarithmic transformation of self-monitoring of blood glucose data^(^ ^49^ ^,^ ^50^ ^)^. The logarithmic transformation is required to give a normal distribution to the otherwise asymmetric glycaemic scale. Indeed, the hyperglycaemic range between 10 and 33 mmol/l (180 and 600 mg/dl) is far greater than the hypoglycaemic range below 4·4 mmol/l (80 mg/dl), and the normal range between 4·4 and 10 mmol/l (80 and 180 mg/dl) is not exactly ‘central’ to the entire possible glycaemic scale. The low blood glucose index (LBGI) and high blood glucose index (HBGI) represent the frequency and extent of low and high blood glucose measurements, respectively. Higher LBGI and HBGI values indicate more frequent or more extreme hypo- and hyperglycaemia, respectively. The LBGI and HBGI can be obtained from both self-monitoring of blood glucose and CGM data and can therefore be used to calculate the blood glucose risk index, LBGI+HBGI, an indicator of the risk of experiencing extreme glycaemic values. The average daily risk range is calculated using 2–4 weeks of self-monitoring data, but requires a glycaemic monitoring frequency of at least three measurements per day^(^ ^51^ ^)^.

In conclusion, many indices of glycaemic variability have been developed, validated and demonstrated to be useful indicators of glycaemia. However, there is no consensus about what could be the standard measure of glycaemic variability for diabetic patients, in part because most measures of glycaemic variability indicate different aspects of glycaemia. Current data on glucose variability among non-diabetics are too scarce to evaluate the best marker in this population that experiences less glycaemic variability.

## Alternative markers of glycaemia and their use in the general population (Eric S. Kilpatrick)

**Fig. 2:**
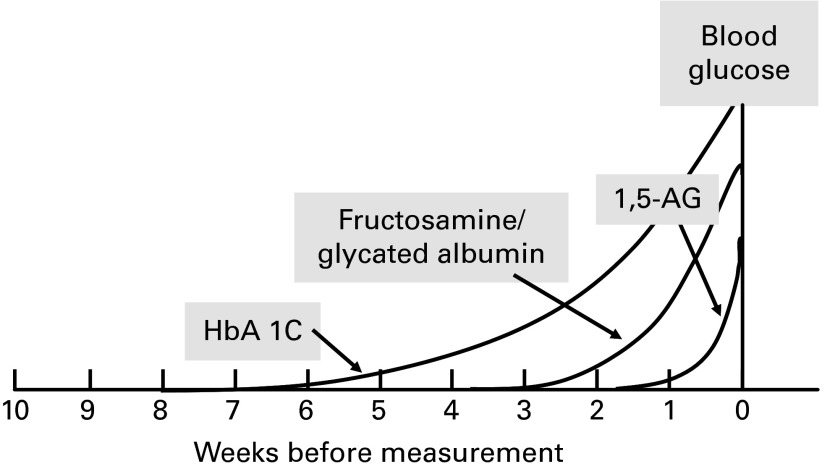
Markers of glycaemic control and their temporary reflection of glycaemic exposure. 1,5-AG, 1,5-anhydroglucitol; HbA1c, glycated Hb.

CGM or multiple within-day measures of pre- and postprandial glucose are the ideal way of assessing glycaemia in both diabetic and non-diabetic populations. However, these measures are laborious, time-consuming and costly, so alternative markers of glycaemia are routinely used in managing patients with diabetes, and these markers can be considered for use in subjects without diabetes^(^ ^52^ ^)^. An important difference among these glycaemic markers is the time frame of previous glycaemic exposure that is reflected (Fig. 2); however, other characteristics of such markers also define their applicability and use.

HbA1c is well established among clinicians as a tool to monitor glucose control, and there is strong evidence of HbA1c being a marker of complications^(^ ^53^ ^)^. HbA1c measures the glycation of Hb and gives an indication of glycaemia over the previous 6–8 weeks, with recent glycaemia having a greater influence on the result than glycaemia in the past^(^ ^54^ ^)^. Between individuals, the same HbA1c value can be associated with a range of mean glucose values. For example, within the non-diabetic range, some subjects with an HbA1c of 31 mmol/mol (5 %) will have a true mean glucose of 4·2 mmol/l, while others will have a mean of 6·7 mmol/l^(^ ^55^ ^)^. However, within the same patient, HbA1c is well placed at tracking changes in their glycaemic control over time. The contribution of postprandial hyperglycaemia to the HbA1c value is still debated, but it would appear that it is unlikely to contribute more to the concentration than would be expected^(^ ^56^ ^)^.

Serum fructosamine is a relatively non-specific marker of serum protein glycation. It gives an indication of glycaemia over a shorter period of time compared with HbA1c (1–3 weeks), which can be an advantage in some clinical situations, such as pregnancy, and where HbA1c may give unreliable results. Glycated albumin assays generally provide a more specific assessment of protein glycation over the same period of time as fructosamine^(^ ^57^ ^)^. In some studies, glycated albumin concentrations have been found to reflect postprandial hyperglycaemia better than pre-prandial, although the mechanism for this remains unclear^(^ ^58^ ^)^. In clinical practice, both fructosamine and glycated albumin are regarded as good alternatives for HbA1c measurement as these markers are unaffected by haemoglobinopathies and anaemia. Limitations for its clinical use are, however, that the treatment targets are not clearly defined and that other factors such as total protein concentrations (fructosamine only) and obesity may have an impact on these levels as well. Glycated albumin and fructosamine may also be the indicators of disease risk as both have been associated with long-term microvascular complications and incidence of diabetes^(^ ^59^ ^,^ ^60^ ^)^.

An indicator of postprandial glycaemia that would appear to be particularly valuable is 1,5-anhydroglucitol (1,5-AG)^(^ ^61^ ^)^, as rises in blood glucose concentrations will lead to glycosuria, which, in turn, increases the urinary loss of 1,5-AG and therefore a fall in serum values. The renal threshold is therefore an important determinant of 1,5-AG, which may limit interpretation in circumstances that affect the threshold (such as pregnancy). The correlation with HbA1c and glucose is rather low^(^ ^62^ ^)^, potentially reflecting the fact that these measures reflect different aspects of glycaemia. Within the non-diabetic range, glycaemia will probably seldom exceed the patients’ renal thresholds for glucose and so 1,5-AG is unlikely to be a useful marker in this group of subjects. The relevance of markers in particular reflecting postprandial hyperglycaemia, among others, lies in the notion that postprandial, more than fasting, glucose levels are generally regarded as a strong risk factor for cardiovascular complications. Although there are currently only a few intervention trials that have studied the cardiovascular effects of lowering postprandial glucose as such^(^ ^63^ ^–^ ^65^ ^)^, these trials have also shown that in diabetic patients, it is difficult to achieve the target for postprandial glucose lowering^(^ ^64^ ^,^ ^65^ ^)^. Nevertheless, many large-scale epidemiological studies have confirmed the prospective association of post-load or postprandial glucose with CVD^(^ ^66^ ^,^ ^67^ ^)^. So far, few data are available on the relationship of 1,5-AG with disease. In the Atherosclerosis Risk in Communities (ARIC) study, 1,5-AG has been shown to be associated with incident diabetes independent of HbA1c and glucose^(^ ^68^ ^)^. More prospective studies on end points are clearly needed to validate the use of 1,5-AG as a marker of disease risk.

CGM is still the optimal approach to fully assess glucose excursions in the diabetic as well as in the non-diabetic population. A new technique measuring glucose in the interstitial fluid is less invasive and provides good estimates of postprandial glucose^(^ ^69^ ^)^. Importantly, it has been proven to be highly acceptable to users and may thus provide a good alternative to established devices^(^ ^69^ ^)^. This approach may be an attractive way forward for evaluating food effects on glycaemic exposure.

In conclusion, although CGM remains the optimal approach to assess glycaemic exposure, routine and less laborious markers have also been extensively used. HbA1c is the best-established marker of glycaemic control in clinical practice. Markers other than HbA1c can be appropriate for use when a measure of shorter duration of glycaemia or glucose variability is sought or when HbA1c is known to be unreliable (e.g. in individuals with haemoglobinopathy, anaemia or end-stage renal disease). The less-invasive novel approach of measuring interstitial glucose may provide an interesting way forward in evaluating glycaemic exposure among individuals with or without diabetes.

## Glycated Hb and advanced glycation end products as markers of glycaemia (Bruce H. R. Wolffenbuttel)

Next to HbA1c, advanced glycation end products (AGE) are long-term markers of glucose control either in the form of Hb-AGE in erythrocytes^(^ ^70^ ^)^ or accumulated in tissues^(^ ^71^ ^)^. In particular, HbA1c but also accumulated AGE have been well validated as the predictors of CVD and other diabetic-related complications^(^ ^72^ ^–^ ^75^ ^)^. Much less is known about the determinants of these markers, which is the topic of the brief review presented below.

HbA1c is a standard measure of glycaemic exposure in clinical practice and has been accepted as a diagnostic criterion for diabetes. A substantial proportion of HbA1c is, however, determined by non-glycaemic factors. In the Lifelines Cohort Study, it has been found in non-diabetic individuals that fasting plasma glucose explains 11 % of the inter-individual variation in HbA1c, with other important contributing factors being non-glycaemic, and the total model explains 26 % of the variance^(^ ^76^ ^)^. Determinants have been demonstrated to be heritable for a large part^(^ ^77^ ^)^, with the heritable part being only to a minor degree glucose-related. Indeed, genome-wide association studies identified ten genetic loci associated with HbA1c. Of these, seven map to loci where common variants probably influence HbA1c levels via erythrocyte biology^(^ ^78^ ^)^. The heritable factor determining HbA1c also became evident from a study comparing HbA1c levels across ethnically diverse groups. It has been found that the level of HbA1c is, in general, approximately 0·3–0·5 % higher in Hispanics and at lower glycaemic levels also in Africans and Asians when compared with Caucasians^(^ ^79^ ^)^.

Tissue AGE accumulation can easily be measured non-invasively by skin autofluorescence, and has also been applied for screening of diabetes^(^ ^71^ ^)^. In diabetic patients, AGE accumulation in the skin has been associated with the duration and severity of hyperglycaemia. Skin autofluorescence measures have been found to be related to age (years), the duration of diabetes, creatinine levels and mean HbA1c over the previous year, but not to the most recent HbA1c measurement^(^ ^71^ ^)^. AGE formed during heating of food can also be partly absorbed and measured in serum, but their contribution to AGE accumulation in tissues and its consequences for health are currently unresolved^(^ ^80^ ^)^. AGE in skin collagen are assumed to provide a longer-term index of tissue damage than HbA1c^(^ ^71^ ^)^. Indeed, Hb-AGE in erythrocytes has been demonstrated to respond more slowly to intensive treatment than does HbA1c^(^ ^70^ ^)^. AGE in skin collagen measured by autofluorescence are regarded as an even more ‘static’ measurement, and it is currently unknown whether this measure can be used for the evaluation of interventions.

In conclusion, both HbA1c and AGE are strong markers of long-term disease risk, but it is evident from human genome-wide association studies, studies in twins and population studies that a substantial proportion of the variation in HbA1c and AGE between individuals is determined by other (non-glycaemic) factors. Where HbA1c reflects glycaemia over approximately 5–6 weeks, AGE accumulation in the skin reflects glycaemia over years. Whether AGE measured by autofluorescence can be used to evaluate the differences within individuals and the effect of ‘interventions’ still needs to be demonstrated.

## Summary of discussion

### Hyperglycaemic exposures in normoglycaemic individuals are limited

It was generally recognised that so far, there is need for more studies because only a few studies have reported continuously measured glucose profiles in non-diabetic individuals over one or more days. Most studies that have averaged group^(^ ^81^ ^)^ or individual^(^ ^82^ ^)^ values over a number of days have shown that the averaged glucose levels generally rise no higher than 7·8 mmol/l in response to meals^(^ ^2^ ^)^. However, it may be expected that individual differences reflecting acute dietary effects are apparent from real-time measures such as CGM. The few recent studies reported on individual hyperglycaemic episodes have indeed indicated that most normoglycaemic individuals do experience certain episodes of hyperglycaemia that exceed the IGT or even the diabetic threshold^(^ ^82^ ^–^ ^84^ ^)^. Thus, although (postprandial) glucose levels in normoglycaemic individuals are for the large majority of the day indeed normoglycaemic, they seem to experience occasional periods of hyperglycaemia in real-life conditions.

### Reductions in glycaemic exposure in the non-diabetic population (e.g. in response to dietary interventions) will not be reflected in every marker that reflects glycaemic exposure

It was assumed that even if certain periods of acute hyperglycaemia do exist in normoglycaemic individuals, variation (e.g. as a result of different diets) in the markers of (sustained) glycaemic exposure would be very small or not apparent in this population. Since ‘normoglycaemic’ implies a well-functioning level of control over both postprandial and fasting glucose, the strongest change in glycaemic markers would be expected when glucose tolerance is lower, or perhaps with interventions that produce large and frequent differences in postprandial glucose. Other factors that will determine the effect size include the type of intervention, assay variability and the nature of the marker (being dynamic or static). It may be expected that 24 h glucose monitoring and glucose levels in the oral glucose tolerance test will be more likely to reflect reductions in glycaemic exposure as these markers are more dynamic than others (e.g. HbA1c). Some markers are not suitable to track changes in glycaemic exposure in the normoglycaemic population; for example, 1,5-AG would only reflect glucose levels that result in glycosuria (9–10 mmol/l), which is unlikely in the non-diabetic population.

### Glycaemic exposure may be a risk factor for progression to disease in an as-yet uncompromised population

The evidence of glycaemic exposure being a risk factor for progression to disease in a healthy population has significant support, but is not conclusive. The strongest evidence comes from studies with acarbose in individuals at risk (IGT)^(^ ^5^ ^)^, while studies on low-GI diets are supportive^(^ ^9^ ^,^ ^10^ ^)^. Nevertheless, as noted, regulatory agencies generally view reduced glycaemic impact of foods as a beneficial physiological effect.

### Appropriate markers of glycaemic exposure are not necessarily appropriate markers of disease risk

There is an important difference between a marker of exposure (reflecting what has already happened) and a marker of risk (predictor of what will happen). Additional data are required to validate the markers of glycaemia as markers of disease risk. HbA1c, fasting glucose and 2 h post-load glucose are widely accepted as the markers of disease risk. More recently, fructosamine, glycated albumin and 1,5-AG have been validated in a few studies as the markers of exposure that may also be markers of disease risk; however, more data are needed to be able to compare different markers.

### Glycated Hb may not be the most appropriate glycaemic exposure marker for validating the efficacy of (dietary) glucose-lowering interventions in the non-diabetic population

HbA1c is certainly most accepted as a glucose exposure marker and (along with fasting blood glucose) benefits from the existence of widely accepted ‘reference’ values. Reference values are less well established for alternative markers, making the clinical relevance of changes in other markers harder to interpret. However, value of HbA1c only reflects glucose levels over a longer time frame and its sensitivity will be limited to individuals with IGT and diabetes.

## Conclusions

The workshop participants agreed that reducing glycaemic exposure is generally regarded as a physiological benefit, and striving to develop dietary interventions and foods that do so is potentially relevant for public health. However, the evidence for a health benefit is so far not conclusive, in particular for the general ‘healthy’ population. Studies with low-GI diets, for example, provide some supportive evidence, but the confounding effects of diet composition make the results difficult to interpret purely in terms of glycaemic exposures. Large intervention trials with acarbose in (pre)diabetes provide the strongest evidence. Studies on biomarkers of heart and liver function suggest that the beneficial metabolic effects of postprandial glucose-lowering interventions may be broad, and these effects could provide relevant mechanistic support for a causal relationship between glycaemic exposure and disease risk. Furthermore, diet-related reductions in postprandial glucose can be achieved via impacts on processes other than just the rate of glucose uptake, which is important for mechanistic understanding.

Overall, workshop participants agreed that markers of glycaemic exposures are sparsely used in intervention studies among non-diabetic populations, and that most data on such markers are derived from diabetic patients. CGM remains the optimal approach to directly assess glycaemic exposure, also in the non-diabetic population. Continuous measurement of ‘interstitial’ glucose has the potential of being an acceptable and less-invasive method in determining changes in glycaemic exposure. Measurement of glycaemic variability also requires CGM. Glycaemic variability can occur independent of overall glycaemic exposure and is a complex concept; different aspects of variability may require different measures. There is no clear consensus about the optimal measures of glycaemic variability among diabetic patients, let alone in non-diabetics.

Routine and less laborious markers of overall glycaemic exposure provide attractive and feasible alternatives to CGM. HbA1c remains the best-established marker that reflects longer-term glycaemic exposure and disease risk in diabetic patients. Alternative markers of glycaemic exposure such as fructosamine, glycated albumin, 1,5-AG and AGE reflect different aspects of glycaemic exposure and can be valuable dependent on the aspect of interest (period reflected and glucose variability). Generally speaking, it appears that markers reflecting longer-term glycaemia such as HbA1c and AGE have the best-established predictive relationship with future chronic cardiometabolic disease risk. For all markers of glycaemia, the responsiveness to intervention will probably be smaller among non-diabetic than among diabetic populations. Further validation and acceptance of glycaemic exposure markers for the general population would aid food innovation and better design of dietary interventions targeting glycaemic exposure.
